# The complex pathophysiology of allergic rhinitis: scientific rationale for the development of an alternative treatment option

**DOI:** 10.1186/s13223-018-0314-1

**Published:** 2019-04-16

**Authors:** Leif Bjermer, Marit Westman, Mats Holmström, Magnus C. Wickman

**Affiliations:** 1 Department of Respiratory Medicine & Allergology, Skane University Hospital, 22185, Lund, Sweden; 2 Dept. of ENT-diseases, Karolinska University Hospital, 171 76 Stockholm, Sweden; 3 Immunology and Allergy Unit, Department of Medicine Solna, Karolinska Institutet, 171 77 Stockholm, Sweden; 4 Dept. of Clinical Science, Intervention and Technology, Division of Ear, Nose and Throat Diseases, Karolinska Institutet, 171 77 Stockholm, Sweden; 5 Department of Environmental Medicine, Karolinska Institutet, 171 77 Stockholm, Sweden; 6 Sach’s Children’s Hospital, 118 83 Stockholm, Sweden

**Keywords:** Allergic rhinitis, Azelastine, Fluticasone, MP-AzeFlu

## Abstract

Allergic rhinitis (AR) poses a global health problem and can be challenging to treat. Many of the current symptomatic treatments for AR have been available for decades, yet there has been little improvement in patient quality of life or symptom burden over the years. In this review, we ask why this might be and explore the pathophysiological gaps that exist within the various AR treatment classes. We focus on the benefits and drawbacks of different treatment options and delivery routes for AR treatments and consider how, given what is known about AR pathophysiology and symptomatology, patients may be offered more effective treatment options for rapid, effective, and sustained AR control. In particular, we consider how a new AR preparation, MP-AzeFlu (Dymista^®^, Meda, Sweden), comprising a formulation of an intranasal antihistamine (azelastine hydrochloride), an intranasal corticosteroid (fluticasone propionate), and excipients delivered in a single spray, may offer benefits over and above single and multiple AR therapy options. We review the evidence in support of this treatment across the spectrum of AR disease. The concept of AR control is also reviewed within the context of new European Union and Contre les Maladies Chroniques pour un VIeillissement Actif-Allergic Rhinitis and its Impact on Asthma initiatives.

## Background

Allergic rhinitis (AR) is a recognised global health problem. It is the most prevalent chronic allergic disease affecting European citizens today, with a prevalence in Sweden of 25% [1]. Although often perceived as a nuisance condition, the reality is AR exacts a high toll on patients’ lives due to considerable symptomatic burden when untreated or undertreated, negatively impacting patients’ quality of life (QoL) [2–7]. AR is felt in all areas of daily living, including performance at work and school [8, 9], and can be associated with poor sleep quality [10], cognitive and mood impairment [11], and even the ability to drive [12].

The burden of AR is also felt socioeconomically, and this, too, is often underestimated and underappreciated [4, 5]. A recent Swedish survey revealed mean annual direct and indirect AR-related costs per individual per year of €210.30 and €750.80, respectively [13]. This amounted to a total annual cost to the economy of €1.3 billion, with presenteeism representing 70% of the total cost [13]. In fact, others have shown that AR has the greatest negative impact on work productivity of any chronic disease, exceeding that of heart disease and diabetes combined [14]. The importance of AR control has now been prioritised at the European Union (EU) level [15].

AR can be challenging to treat. Most patients presenting to their physician have moderate/severe disease [6, 16, 17], and many experience persistent symptoms [18] and are polysensitised [8, 19]. Difficult to treat phenotypes have emerged, including mixed rhinitis [both AR and non-AR (NAR)] [20], severe chronic upper airway disease (i.e. uncontrolled disease despite guideline-directed care) [21, 22], and local AR (i.e. localised nasal allergic response in the absence of systemic atopy) [23–25]. More effective pharmacological treatments are needed to meet these challenges.

There are currently many AR treatments available, with antihistamines, leukotriene receptor antagonists (LTRAs), oral steroids, and intranasal corticosteroids (INSs) recommended in a step-wise approach according to AR phenotype and severity [5, 26]. INSs are still considered the most effective pharmacological AR treatment option; newer ones with lower systemic bioavailability have been introduced, but with no differences between them in therapeutic effect [27, 28]. These symptomatic AR treatments have been available for decades, but despite this, there has been no substantial improvement in patients’ QoL or symptom burden [29, 30]. One of the most recent additions to the AR armamentarium is MP-AzeFlu (Dymista^®^, Meda, Sweden), a novel formulation of an intranasal antihistamine [INAH; azelastine hydrochloride (AZE)] and an INS [fluticasone propionate (FP)] in a single spray [31]. It is recommended as a first-line treatment option for AR patients with a visual analogue scale (VAS) score < 5 cm and as a preferred treatment for those with a VAS score > 5 cm according to the updated allergic rhinitis and its impact on asthma (ARIA) treatment guideline, the AR clinical decision support system (CDSS; use of a simple VAS to assess AR control is discussed later in this text) [32]. Recently published Spanish guidelines also position MP-AzeFlu as a first-line treatment for moderate-severe AR, in preference to an INS for moderate-severe persistent AR [33], and the 2016 update to the ARIA guidelines recommends this combination for seasonal AR (SAR) [4].

In terms of current AR management, there are two major gaps. The first is the “pathophysiological gap”. AR patients continue to experience symptoms despite single- and multiple-therapy AR treatment regimens [17, 34]. The second is the “control gap”, defined as lack of a common AR control concept and language. The aim of this review is to evaluate these gaps and show how recent pharmacological and control-assessment advances help to narrow them.

### The complex pathophysiology of allergic rhinitis: a crash course

**Fig. 1:**
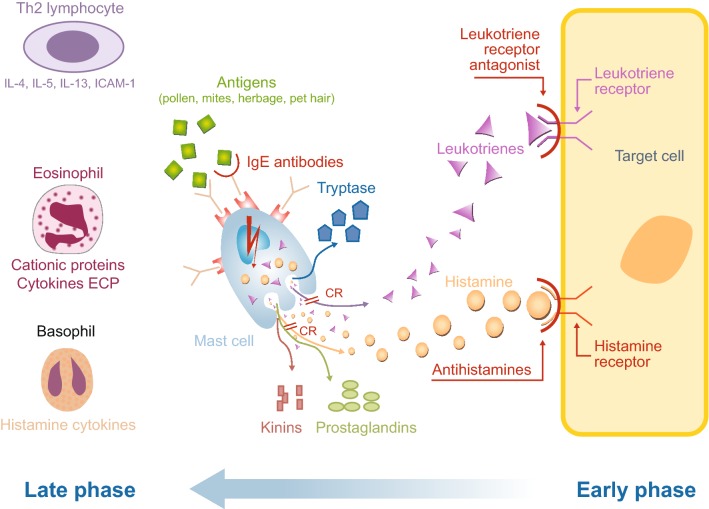
Early and late phases showing the pathophysiological processes and drivers of allergic rhinitis and the potential sites for pharmacological intervention. *ECP* eosinophil cationic protein, *ICAM*-*1* intercellular adhesion molecule-1, *IgE* immunoglobulin E, *IL* interleukin

The pathophysiology of AR is complex, comprising an early- and late-phase allergic response [35, 36]. The process is triggered by exposure to allergens such as pollen, mites, and/or animal dander that are recognised by antigen-specific immunoglobulin E (IgE) receptors on mast cells and basophils in presensitised individuals. The early-phase reaction is characterised by mast cell degranulation (Fig. 1). This phase is associated with the rapid onset (over a period of minutes) of acute nasal symptoms (i.e. sneezing and rhinorrhoea) and the emergence of ocular symptoms (i.e. itching, redness, and watering). These symptoms are caused by histamine release, particularly from mast cells in the nasal mucosa. This early-phase histamine release, together with the effects of other potent pro-inflammatory cytokines (e.g. leukotrienes) and eicosanoids (e.g. prostaglandins and kinins) also increases vascular permeability, leading to oedema formation.

The late-phase reaction develops over a period of hours after exposure to an allergen. It is characterised by cellular recruitment of basophils, neutrophils, T-lymphocytes, monocytes, and eosinophils, and by the release of multiple mediators, including cytokines, prostaglandins, and leukotrienes, which perpetuate the inflammatory response [35, 36]. This late-phase inflammatory reaction is associated with tissue remodelling, further tissue oedema, and the development and perpetuation of nasal congestion, considered by patients to be one of the most troublesome symptoms of AR [37, 38]. As a result of mucosal inflammation, tissues become primed and react more vigorously to allergen exposure. These late-phase reactions and modifications in tissue responsiveness contribute to bronchial hyper-responsiveness (Fig. 1).

### Pathophysiology: impact on AR symptoms and human burden of disease

Nasal and ocular AR symptoms, associated with both early and late response, can have an adverse impact on patients’ QoL and daily functioning [39–41]. When allergy symptoms are at their worst, on a near-daily basis, patients with AR may feel tired, miserable, and irritable at least some of the time [42]. The duration and severity of ocular symptoms such as itchy and watery eyes, eyelid oedema, and asthenia have a particularly high impact on QoL, even greater than that of nasal obstruction and pruritus [17]. A survey conducted in the United States, Latin America, and the Asia-Pacific region revealed that 35–50% of adult AR patients reported nasal symptoms had at least a moderate effect on their daily lives [29]. Another survey in the United States found that patients with AR were twice as likely to report limitations on daytime physical indoor and outdoor activities than those without nasal allergies [43]. The negative impact on daily activities for patients with AR can be greater than that for patients with type 2 diabetes and hypertension [44].

Nasal congestion is the hallmark of the allergic response. This symptom is associated with sleep-disordered breathing, a condition that can have a profound effect on productivity and increased daytime sleepiness [45]. More than 80% of AR and NAR patients report impaired sleep quality linked with nasal obstruction [46]. Approximately one in four adult patients report they are unable to sleep or are awakened during the night because of their symptoms [29], whereas up to 45% of children experience sleep disruption because of nasal allergy symptoms [42]. Furthermore, many of the key pathophysiological mediators of AR responses (i.e. histamine, leukotrienes, cytokines, and prostaglandins) play a role in sleep regulation and might be directly involved in this feature of the condition, independent of nasal obstruction [47].

### Mode of action of allergic rhinitis pharmacotherapies

Pharmacotherapies interact with the AR pathophysiological pathway at different points, reflecting their different modes of action (Fig. 1).

#### Leukotriene receptor antagonists

LTRAs (e.g. montelukast, zafirlukast, and pranlukast) block the activity or secretion of cysteinyl leukotrienes (CysLTs), a potent inflammatory mediator associated with nasal congestion, mucus production, and inflammatory cell recruitment that typifies AR [48]. They also have other anti-inflammatory activities, including inhibition of 5-lipoxygenase [49–51], histone acetyltransferase [52], adenosine 3′,5′-cyclic monophosphate phosphodiesterase [50], eosinophil migration, adhesion to vascular endothelium [53], and nuclear factor kappa-light-chain-enhancer of activated B cell-activation [54, 55]. Additionally, LTRAs can inhibit reactive oxygen species generation, as well as the release of protease and elastase from isolated human neutrophils [50, 56]. However, whether these non-CysLT-mediated effects are physiologically relevant in AR or achievable at clinical drug concentrations remains to be determined.

#### Oral antihistamines

At concentrations achievable in vivo, oral antihistamines (OAHs; e.g. loratadine, desloratadine, cetirizine, and levocetirizine) competitively inhibit the interaction of histamine with H_1_ receptors. At high micromolar concentrations, OAHs may also inhibit histamine-stimulated cytokine release and antagonise or inhibit other mediators of early- and late-phase allergic reactions to various degrees [57–62]. Whether such high concentrations are achievable in the nasal mucosa following oral administration of these drugs is unlikely [63, 64]; however, it is likely that the desired concentrations to affect these additional anti-allergic actions are achievable with an INAH [65].

#### Intranasal antihistamines

Intranasal application of antihistamines (e.g. AZE, olopatadine, and levocabastine) ensures delivery of active drug directly to the nasal mucosa, thus enhancing its local anti-allergic and anti-inflammatory effects, while minimising systemic exposure to therapy [65]. Many studies have shown the efficacy, safety, and tolerability of INAHs for the treatment of AR [65]; however, the majority of them relate only to AZE. AZE has multimodal action in AR, displaying not only H_1_-antagonist activity but also mast cell-stabilising, anti-leukotriene, and anti-inflammatory effects [65]. Its antagonism at the H_1_ receptor effectively neutralises histamine-induced increases in vascular permeability and capillary fluid leakage responsible for rhinorrhoea, nasal pruritus, and sneezing [66, 67]. Mast cell stabilisation occurs as a consequence of AZE, blocking the IgE-regulated calcium channels on mast cells that are involved in mast cell degranulation, thus preventing histamine and other mast cell mediator release (e.g. tryptase, prostaglandins, kinins, and interleukins) [68, 69]. Anti-leukotriene effects occur due to prevention of leukotriene release from mast cell degranulation, reduction of leukotriene (LT)B_4_ and LTC_4_ production, and inhibition of phospholipase A2 and LTC_4_ synthase [70]. AZE’s anti-inflammatory properties stem from a reduction in the number of inflammatory cells and mediators. These include eosinophils and neutrophils and mediators such as tumour necrosis factor (TNF)α [71], granulocyte macrophage colony-stimulating factor (GM-CSF), cytokines (e.g. interleukin [IL]-1β, IL-4, and IL-8) [72, 73], and adhesion molecules (e.g. intercellular adhesion molecule-1 [ICAM-1]) [74], all of which perpetuate the inflammatory response and induce symptoms of the late-phase response (i.e. congestion, rhinorrhoea, and hypersensitivity) [68]. Research into the comprehensive pharmacological profile of AZE continues, with a recent study showing that AZE interferes with a specific immune interaction between T cells and dendritic cells in vitro [75].

#### Intranasal corticosteroids

INSs are potent inhibitors of the late-phase allergic reaction in AR. They inhibit recruitment of Langerhans cells, macrophages, mast cells, T cells, and eosinophils into the nasal mucosa [76–79]. This is most likely due to reduction of ICAM-1 expression on nasal epithelial cells, both during the early and late phases after allergen challenge [80]. INSs may also prevent or reduce airway remodelling via downregulation of nasal fibroblast functions [81]. INSs potently inhibit inflammatory mediator release from many cells involved in the pathophysiology of AR. For example, they have been shown to reduce concentrations of IL-4, IL-5, and IL-13 in nasal secretions following allergen challenge [82], and to suppress release of interferon-γ, IL-2, and IL-17 after allergen challenge in vitro models of human nasal mucosa [83]. In vitro studies indicate that INSs can reduce IL-13 production in mitogen-stimulated cells [84] and reduce production of GM-CSF, TNFα, IL-6, and IL-8 generated by cultured nasal epithelial cells [85, 86]. The broad anti-inflammatory actions of INSs may prevent or reduce airway hyper-reactivity in chronic AR, and treatment with an INS has been shown to objectively reduce nasal congestion and nasal histamine hyper-reactivity in children and adolescents with perennial AR (PAR) [87].

### Pathophysiological gaps: location and clinical consequences

The pharmacological profiles of available treatments for AR show that there are a number of “pathophysiological gaps” within the AR pharmacopeia (Fig. 1). For example, at concentrations achieved following clinical dosing, LTRAs are likely to be active predominantly at the leukotriene receptor [88], whereas OAHs at physiological concentrations will be active predominately at the H_1_ receptor [62, 89]. Although AZE offers a broad mechanism of action, it does not inhibit mast cell recruitment or interfere with leukotriene receptor interactions [65]. Likewise, INSs do not inhibit mast cell degranulation or directly interrupt leukotriene and histamine receptor interactions [90].

As a consequence, no single medication class is capable of providing rapid and complete relief from all symptoms associated with AR (Table 1) [5, 91]. LTRAs and OAHs both act at a single site on the AR pathophysiological pathway and appear to have broadly similar efficacy [92]. Even so-called new-generation OAHs, such as rupatadine, a dual blocker with anti-H_1_ antagonist properties and inhibitory effects on platelet activating factor [93], may not offer a sufficiently broad-spectrum mechanism of action to affect key pathophysiological drivers of AR. Studies have found efficacy advantages of rupatadine over other OAHs [94, 95], but not over INAHs [96]. AZE, by virtue of its topical application and multimodal mechanism of action [65], provides superior congestion and sneezing relief over OAHs [97]. It also provides superior ocular symptom relief to that of INSs and similar relief from nasal pruritus and sneezing to that of INSs [91]. However, INSs, as the most potent anti-inflammatory agents, offer superior relief from congestion and rhinorrhoea over AZE [91]. The areas in which INSs seem to fall short are their inconsistent effects on the eye [98] and the length of time they take to reach maximal effect.

**Table 1:** Overview of the relative effectiveness of treatment classes used in the management of AR: assessment of effectiveness in controlling symptoms of AR

Symptom	Leukotriene antagonist	Oral antihistamine	Intranasal antihistamine	Intranasal corticosteroid
Nasal congestion	+	±	+	++
Nasal pruritus	+	+	+	+
Rhinorrhoea	+	+	+	++
Sneezing	+	+	++	++
Ocular itching	±	±	++	+
Ocular watering	±	±	++	+
Ocular redness	±	±	++	+

Informed by allergic rhinitis and its impact on asthma (ARIA) guidelines [5] and Carr et al. [91]

*AR* allergic rhinitis

++: especially effective, +: effective, ±: mixed efficacy

### Ocular symptom control: the pathophysiological gap

A range of ophthalmic agents are available for control of AR-associated ocular symptoms; however, their use is limited by poor tolerance for eye drops among many patients, difficulty of administration in a sterile manner, the need for frequent application, and lack of patient compliance [99]. Thus, patients may need to use INS and OAH for ocular symptom control. Notwithstanding the time course required for efficacy, a number of reviews report that INSs, as a class, have a positive impact on ocular symptoms in AR, improving combined total eye symptom scores, as well as individual symptoms such as redness, itching, tearing, and oedema [100–102]. It is speculated that this effect is mediated through the naso-ocular reflex; however, it should be noted that not all INSs are equally consistent in managing the ocular symptoms of AR, with fluticasone outperforming mometasone in this regard [98, 103]. Furthermore, proof of the ocular efficacy of INSs comes from comparison with placebo, rather than with active therapy [104–106].

OAHs inhibit some symptoms of pruritus and erythema in laboratory models and have been shown to be superior to placebo in ocular symptom relief in clinical trials. In general, OAHs are considered superior to INSs in relief of ocular symptoms, but some studies have shown equal efficacy with these agents or even superior efficacy with INSs [107]. However, OAHs, like INS, do not provide a sufficiently speedy ocular response. A combination of topical application and the multimodal effects of INAHs mean that these agents offer effective and rapid ocular symptom relief. For example, more patients treated with intranasal AZE achieved substantial ocular symptom relief than those on FP, and they achieved this response many days faster [91].

### Pathophysiological gaps: what does this mean in practice?

Pathophysiological gaps associated with the various AR treatment options mean that many patients remain symptomatic despite treatment [6, 17, 19, 21, 108–110]. For example, patients with moderate-severe AR in the United Kingdom have reported significant nasal and ocular symptom burden, despite the fact that most of them were actively treated (96.2%), with 70.5% on multiple therapies [34]. Moreover, these patients reported significant AR-associated absenteeism (4.1 days/year) and presenteeism, with a negative impact on productivity of more than 50% 38 days out of the year.

However, increasing the dose of the INS is not the answer, owing to its flat dose–response curve. Switching from one INS to another is also not the answer, since available INSs have comparable efficacy [27, 28]. Although adding on oral therapies (e.g. OAHs or LTRAs) might seem like a logical step, this approach offers little or no additional benefit over an INS alone in terms of control of nasal and ocular symptoms [111–113], and in the past was not recommended by ARIA due to insufficient evidence [5, 26]. The 2016 update to the ARIA guidelines does suggest (with low to moderate certainty) that combination treatment with an OAH or INAH and an INS may be appropriate for patients with SAR [4]. Concurrent use of an INS and INAH has provided benefits over monotherapy in patients with moderate-severe SAR [114]; however, significant problems with this approach have been noted, including a negative impact on concordance, increased runoff both posteriorly and anteriorly [115], and nonhomogeneous distribution of active agents on the nasal mucosa [116]. All of these factors highlight the need to simplify AR treatment, with a single medication option and with broader pathologic coverage than OAHs, INSs, and LTRAs, providing better and faster symptom control.

### MP-AzeFlu: an option for filling some pathophysiological gaps

MP-AzeFlu comprises an INAH (AZE), an INS (FP), and a novel formulation in a single spray. It therefore offers the benefits of broad pathophysiological coverage due to incorporation of two agents from different medication classes with different yet complementary modes of action, antagonises both early- and late-phase allergic responses, and is convenient to use. Each of MP-AzeFlu’s components (i.e. AZE, FP, and formulation) contribute to its superior clinical effect and effectiveness over currently considered first-line therapies for AR [117], providing significant additive effects in many instances [118, 119].

### MP-AzeFlu: preclinical evidence

#### Eosinophil survival

A recent study investigated the anti-inflammatory effect of MP-AzeFlu compared with AZE and FP alone in a validated in vitro model of eosinophilic inflammation [119]. MP-AzeFlu had an inhibitory effect on IL-6 secretion, significantly stronger than that induced by FP or AZE alone, compared with control values (*p* < 0.05). Furthermore, the inhibitory effect of MP-AzeFlu at a 1:10^2^ dilution on eosinophil survival at days 3 and 4 was more than twice that induced by FP or by AZE (*p* < 0.05).

#### Improved delivery, retention, and distribution at the nasal mucosa

Intranasal sprays must be delivered to the nasal cavity in sufficient volume, at appropriate viscosity and droplet size, and with a technique that allows optimal retention, maximises absorption from the mucosa, and has the potential for maximum therapeutic effect. It is unlikely that concurrent use of intranasal FP and AZE would provide the same benefits as MP-AzeFlu, for two reasons. One, it is unlikely that patients would comply with a double intranasal therapy regimen in the long term, most likely choosing to use one spray only. Two, the clinical use of sequential FP and AZE requires dosing 948 μl in contrast with 274 μl for MP-AzeFlu. Application of larger volumes (more than 300–400 μl) of fluid can result in significant nasal drip (run off), as demonstrated in a human nasal cavity in vitro model [115]. Application of MP-AzeFlu in a single spray provided more uniform distribution and greater retention in the nasal cavity than sequential sprays of AZE and FP (either branded or generic) [116]. Both runoff and uneven active agent distribution could diminish efficacy.

### MP-AzeFlu: clinical evidence

The effect of MP-AzeFlu on both the early- and late-phase allergic response may be observed clinically. An overview of the clinical development programme devised to study MP-AzeFlu is shown in Table 2 [118, 120–130].

**Table 2:** Overview of MP-AzeFlu’s clinical development programme

Study (NCT or EudraCT number)	Duration	AR phenotype	*N* (population)	Comparator(s)	References
*Pharmacokinetic studies*					
3282	Single dose	N/A	19 (PP)	Marketed FP and FP in the MP-AzeFlu-based formulation and device	[122]
3283	Single dose	N/A	26 (PP)	Astelin^®^ and AZE in the MP-AzeFlu formulation and device	
*Randomised clinical trials*					
Adults and adolescents					
MP4001 NCT00660517	14 days	SAR	607 (ITT)	Marketed comparators Astelin and generic FP	[118, 125]
MP4002 NCT00651118	14 days	SAR	831 (ITT)	Regulatory comparators AZE and FP in the MP-AzeFlu formulation and device	[121]
MP4004 NCT00740792	14 days	SAR	776 (ITT)		
MP4006 NCT00883168	14 days	SAR	1791 (ITT)		
MP4000 EudraCT 2011-001368-23	52 weeks	PAR and NAR	611 (safety)	Marketed FP	[128, 129]
Children (aged ≥ 4 to 12 years)					
MP4007 NCT01794741	12 weeks	AR	405	Marketed FP	[120]
MP4008 NCT01915823	14 days	SAR	348	Placebo	[130]
*Real-life study*					
NIS 3299	≈ 14 days	SAR and PAR	Germany: 1781 Sweden: 431 Denmark: 170 Norway: 159	None	[123, 124, 126, 127]

*AR* allergic rhinitis, *AZE* azelastine hydrochloride, *FP* fluticasone propionate, *ITT* intent to treat, *MP*-*AzeFlu* a novel formulation of an intranasal antihistamine, azelastine hydrochloride, and an intranasal corticosteroid, fluticasone propionate, in a single spray, *N/A* not applicable, *NAR* non-AR, *NIS* noninterventional study, *PAR* perennial allergic rhinitis, *PP* per protocol, *SAR* seasonal allergic rhinitis

#### Pharmacokinetic studies

Pharmacokinetic studies showed MP-AzeFlu’s formulation and device had no impact on AZE bioavailability, but did increase FP bioavailability [122]. The bioavailability of AZE was comparable to a reformulated AZE preparation (i.e. AZE in the MP-AzeFlu formulation and device) and commercial AZE, and there was no evidence of drug–drug interactions [122]. Although an increase in serum concentrations of FP was observed for MP-AzeFlu and for FP in the MP-AzeFlu formulation and device compared with marketed FP [122], it should be noted that serum FP levels were low for all investigational products and elevations were not considered clinically meaningful (elevations of a factor of eight would be needed to affect safety) [131].

#### Controlled clinical studies in adults

The efficacy and safety of MP-AzeFlu was assessed in four 14-day SAR studies [118, 121]. The first of these studies compared MP-AzeFlu with a commercially available INS and INAH [118]. The other studies compared MP-AzeFlu with formulation- and device-matched AZE and FP (i.e. not commercially available), in order to eliminate the well-known effect of formulation [132]. When this effect was removed, MP-AzeFlu still provided significantly greater overall nasal symptom relief than either FP, AZE, or placebo, with a relative difference of 30% and 39% to FP and AZE, respectively [121]. Patients treated with MP-AzeFlu also experienced significantly superior relief from their ocular symptoms than those treated with FP alone [121]. Also, more MP-AzeFlu-treated patients achieved a 50% reduction in their overall nasal symptom burden and complete or near-to-complete symptom relief, and they did so many days earlier than those treated with FP or AZE [121].

**Fig. 2:**
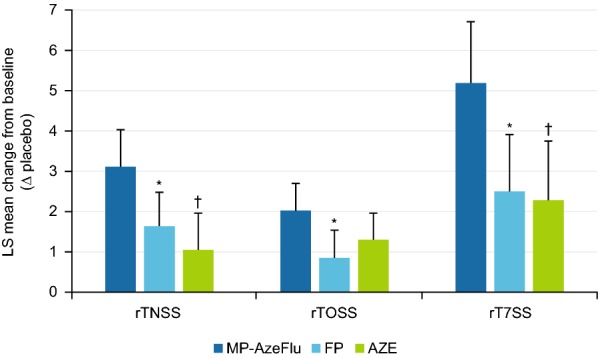
Change from baseline in reflective total nasal symptom score (rTNSS), reflective total ocular symptom score (rTOSS), and reflective total of seven symptom scores (rT7SS) following 14 days’ treatment with MP-AzeFlu (n = 153), fluticasone propionate (FP; n = 151), or azelastine hydrochloride (AZE; n = 152). *LS* least squares, *MP*-*AzeFlu* a novel formulation of an intranasal antihistamine, azelastine, and an intranasal corticosteroid, fluticasone propionate, in a single spray. The precision of these estimates is indicated by the upper bounds of the respective 95% confidence intervals. *p ≤ 0.0031 versus MP-AzeFlu. ^†^*p* ≤ 0.0004 versus MP-AzeFlu (Modified from Meltzer et al. [118])

The treatment difference was greater compared with commercially available FP (i.e. Flonase^®^ generic) and AZE (i.e. Astelin^®^) [118]. In this case, a relative difference of 47% to FP and 66% to AZE was noted for nasal symptoms and a relative difference of 58% and 35% to FP and AZE, respectively, was noted for ocular symptoms. When nasal and ocular symptom scores were combined, MP-AzeFlu was more than twice as effective as either FP or AZE (Fig. 2) [118]. Over-additive effects of MP-AzeFlu were observed for those patients who had particularly bothersome nasal symptoms, most notably nasal congestion, and ocular itching. The analysis also revealed that more patients treated with MP-AzeFlu achieved a halving of their nasal symptom burden (one in every two patients) and complete or near-to-complete response, and they did so about a week faster than those treated with either FP or AZE [118]. This time advantage of MP-AzeFlu over FP and AZE is clinically relevant since an average moderate-severe SAR symptom episode lasts 12.5 days [34]. MP-AzeFlu was well tolerated in all four SAR randomised controlled trials [118, 121].

The contribution of formulation to the efficacy of MP-AzeFlu may be estimated by comparing the results of the study in which the effect of formulation was eliminated [121] with the results of the study in which it was not [118]. A greater treatment difference was observed in the latter. These findings are summarised in Table 3 [118, 121].

**Table 3:** Contribution of MP-AzeFlu’s formulation to its superior efficacy over fluticasone propionate (FP), measured for reflective total nasal symptom score (rTNSS), reflective total ocular symptom score (rTOSS), and time to achieve ≥ 50% reduction from baseline in rTNSS

	Study MP4001 [118] (effect of formulation included)			Studies MP4002/MP4004/MP4006 [121] (effect of formulation removed)			Formulation effect
Parameter	MP-AzeFlu n = 153	Marketed FP n = 151	MP-AzeFlu–FP	MP-AzeFlu n = 848	Reformulated-FP n = 846	MP-AzeFlu–FP	
LS mean rTNSS (95% CI)	− 5.31	− 3.84	− *1.47* (− *2.44*, − *0.50*) *p* = *0.003*	− 5.56	− 4.80	− *0.76* (− *1.18*, − *0.34*) *p *= *0.0004*	*0.71 points*
LS mean rTOSS (95% CI)	− 3.33	− 2.17	− *1.17* (− *1.91*, − *0.42*) *p *= *0.002*	− 3.18	− 2.71	− *0.47* (− *0.78*, − *0.16*) *p* = *0.0030*	*0.71 points*

	Patients at day 14, %		No. days’ advantage over FP	Patients at day 14, %		No. days’ advantage over FP	
≥ 50 reduction from baseline rTNSS	49.1	38.2	≤ *6 days* *p *= *0.0284*	~ 50	≈ 45	≤ *3 days (significance not provided)*	≤ *3 days*

*LS* least squares, *MP*-*AzeFlu* a novel formulation of an intranasal antihistamine, azelastine hydrochloride, and an intranasal corticosteroid, fluticasone propionate, in a single spray

**Fig. 3:**
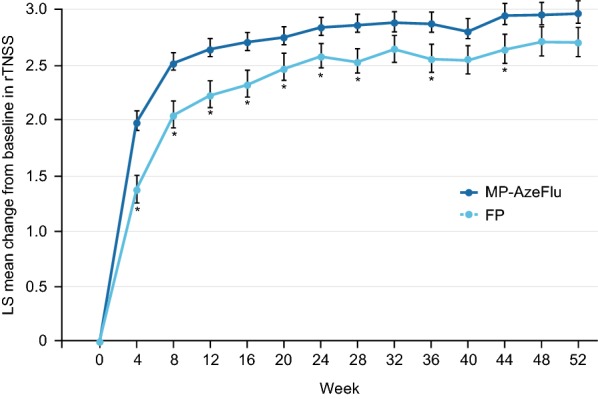
Effect of MP-AzeFlu (n = 388, blue) and fluticasone propionate (FP; n = 199, orange) on reflective total nasal symptom score (rTNSS) in patients with chronic rhinitis. Data are presented as least squares (LS) mean change from baseline at 4-week intervals. *MP*-*AzeFlu* a novel formulation of an intranasal antihistamine, azelastine hydrochloride, and an intranasal corticosteroid, fluticasone propionate, in a single spray, *rTNSS*, reflective total nasal symptom score. *p ≤ 0.0453 versus MP-AzeFlu (Adapted with permission from the* Journal of Investigational Allergology and Clinical Immunology*)

MP-AzeFlu also demonstrated a significant and speedy reduction in overall nasal symptoms compared with marketed FP in an open-label study of patients with chronic rhinitis (i.e. PAR or NAR) [129]. Statistical superiority over FP was noted from day 1 and maintained up to and including week 28, with treatment difference sustained for 52 weeks (Fig. 3) [129]. Approximately seven of ten patients treated with MP-AzeFlu experienced complete symptom relief in the first month of treatment and did so a median of 9 days faster than patients treated with FP. Over the whole year patients treated with MP-AzeFlu experienced 26 more symptom-free days than FP-treated patients (8.4% more; *p* = 0.0005) [129]. A similar pattern was observed for PAR [129].

MP-AzeFlu was well tolerated in clinical trials, with no safety findings that would preclude its long-term use [128]. The most commonly reported treatment-emergent adverse events were those usually reported for AZE (i.e. dysgeusia) and FP (epistaxis and headache) and were considered mild in severity in majority of cases [121, 125]. Dysgeusia was reported as a mild deviation in taste and occurred at approximately the same rate as in AZE monotherapy [133]. Mucosal erosion, identified as superficial and mild in severity requiring no treatment, was reported in one trial [134].

#### MP-AzeFlu: efficacy and safety in children

**Fig. 4:**
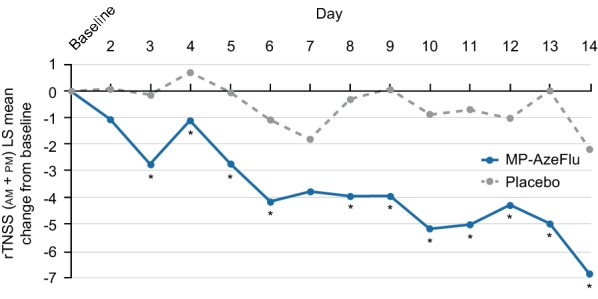
Least squares (LS) mean change from baseline in reflective total nasal symptom score (rTNSS; am + pm) per day when most children (n = 82; > 90%) rated their own symptoms following treatment with MP-AzeFlu or placebo, both one spray per nostril twice daily, for 14 days in children aged 6–11 years with moderate-severe SAR. *MP*-*AzeFlu* a novel formulation of an intranasal antihistamine, azelastine hydrochloride, and an intranasal corticosteroid, fluticasone propionate, in a single spray, *SAR* seasonal allergic rhinitis. *p ≤ 0.040 versus placebo (Reprinted with permission from Berger et al. [130])

The efficacy and safety of MP-AzeFlu in paediatric AR has also been assessed [120, 130]. Children (aged 4–12 years) with SAR treated with MP-AzeFlu for 2 weeks experienced a statistically superior and clinically relevant improvement in their QoL compared with those who received placebo. Improvement was also observed in overall and individual nasal symptom relief, with a greater treatment effect noted as the degree of self-rating increased (Fig. 4) [130]. A simplified symptom scoring system was utilised to assess efficacy in a second study; when all children assessed their own symptoms (rather than their caregiver, by proxy), MP-AzeFlu provided significantly greater AR symptom relief than FP [120]. MP-AzeFlu was well tolerated in both studies [120, 130]; the most common treatment-related adverse event was dysgeusia, which was considered by investigators to be mild in severity [130]. MP-AzeFlu has been granted approval for use in this age group by the US Food and Drug Administration [135].

### The control gap: what it is and how to fill it

The aim of treatment is to achieve and maintain disease control, but there is a lack of consensus on what “AR control” actually is. The concept of control is well defined for other respiratory conditions such as asthma [136] and categorised as well controlled, partly controlled, and uncontrolled. However, in AR and rhinosinusitis the control concept is in its infancy [108]. The importance of AR control has now been prioritised at the EU level [15]. Attempts to define AR control are hampered by the lack of a common language to describe it. The reflective total nasal symptom score (rTNSS) and Rhinitis Quality of Life Questionnaire (RQLQ) are considered gold-standard efficacy assessments, but what do changes in these scores mean to patients, and how might we best fill the control gap that exists for people who continue to suffer the symptoms and impact of AR?

Many experts believe that a simple tool to both assess and define control is needed [32, 137]. In common with other disease states, the VAS can offer a potentially relevant and simple tool to assess control and response to treatment. The VAS appears suited to this task as it correlates well with rTNSS and overall RQLQ score [138] and is sensitive enough to discriminate according to disease severity [139] and to assess the efficacy of AR treatments [140, 141]. In view of this, Contre les MAladies Chroniques pour un VIeillissement Actif (MACVIA)-ARIA (an EU reference site on active and healthy aging) proposed a new treatment algorithm (called AR CDSS) [32] utilising a simple VAS to determine AR control and incorporated this VAS into a free smart phone app for patients (called *Allergy Diary*) [137]. *Allergy Diary* uses the VAS to track symptoms daily and categorise disease as well controlled, partly controlled, or uncontrolled according to predefined score cutoffs, thereby encouraging patients to take their AR medication and seek medical advice when appropriate. The AR CDSS uses a VAS score cutoff of 50 mm to assess control and guide treatment decisions [32]. The overall aim of these tools is to facilitate better communication between all stakeholders involved, allowing doctors to more easily comply with guidelines. Such tools can be used in the controlled clinical trial setting as well as in the real world.

### Assessing AR control using a visual analogue scale

To date, the literature reporting use of VAS scores to quantify and assess the burden of AR and the impact of treatments is relatively sparse. The studies of OAHs, INSs, and LTRAs assess their effect on symptom severity. These studies were done prior to the introduction of the MACVIA-ARIA concept of disease control [32] and therefore use different VAS questions and VAS anchors. However, the VAS studies of MP-AzeFlu do examine the effect on disease control as advocated by MACVIA-ARIA.

#### Studies of oral antihistamines

Bousquet and colleagues [141] assessed the efficacy of desloratadine [5 mg once daily (QD)] in patients older than 12 years with intermittent AR using a VAS to score symptom severity from 0 mm (not all bothersome) to 100 mm (very bothersome). Desloratadine treatment was associated with a significant reduction in VAS score from 57.4 mm at baseline to 40.2 mm after 2 weeks, a shift of 17.2 mm. This reduction in VAS score failed to reach the clinically relevant threshold, previously defined as a VAS score change of at least 23 mm [16]. Those in the placebo group experienced a VAS score reduction of 10.9 mm over the same time period [141]. A follow-up article on those with persistent AR reported that patients treated with desloratadine for 12 weeks experienced a 32.5 mm reduction in VAS score (approximated from graph), compared with a 29 mm reduction experienced by the placebo group [140]. However, significance versus placebo was lost by week 10, and unfortunately, only change from baseline in VAS scores was provided rather than actual scores.

#### Studies with intranasal corticosteroids

Ratner et al. [142] compared the effectiveness of a 15-day course of intranasal FP (200 μg QD) with oral montelukast (10 mg QD) in 705 patients with SAR, using four separate VASs for each of the four most common AR nasal symptoms (i.e. congestion, rhinorrhoea, sneezing, and itching). These VASs were anchored from 0 (no symptoms) to 100 (maximum symptom severity). FP treatment provided significantly better nasal symptom relief, associated with an approximately 30 mm VAS score reduction for each of the four nasal symptoms (averaged over weeks 1–2) compared with an approximately 20 mm VAS score reduction for montelukast (*p* < 0.001 for each symptom) [142]. However, again the authors reported change from baseline in VAS scores, rather than actual scores. Furthermore, calculation of the total symptom score was done by adding each of the individual symptom VAS scores together (rather than having a separate VAS for total symptom score), making it difficult to compare these results with those of other VAS studies. Other studies have used a VAS to compare the effectiveness of INS, but for their effects on hyposmia and nasal discharge in PAR patients, rather than on symptom severity [143].

#### Studies with leukotriene receptor antagonists

Yamamoto et al. [144] assessed whether add-on loratadine might be effective for SAR patients showing unsatisfactory control of symptoms with montelukast during a pollen season. They used a VAS to assess unsatisfactory control (i.e. VAS score > 50 mm) and measure symptom severity reduction for total symptoms and each of the four usual AR individual nasal symptoms from 0 (no symptoms) to 100 (highest severity symptoms). There was some evidence to show that adding loratadine to montelukast was beneficial in alleviating the symptoms of sneezing and rhinorrhoea versus montelukast alone. Patients showed a mean rhinorrhoea baseline VAS score in both groups of approximately 43 mm (at pollen season peak), which reduced to 25.3 mm in the combination group compared with 41.1 mm in the montelukast-only group (*p* < 0.005; midpollen season), and ended at approximately 20 mm for both groups by season’s end [144]. A similar pattern was observed for sneezing VAS scores over the same period. Unfortunately, as with other studies, the exact wording of the question posed to patients prior to completing each VAS was not published. Additionally, the method by which total symptom VAS scores were calculated was not provided.

#### Studies with MP-AzeFlu

**Fig. 5:**
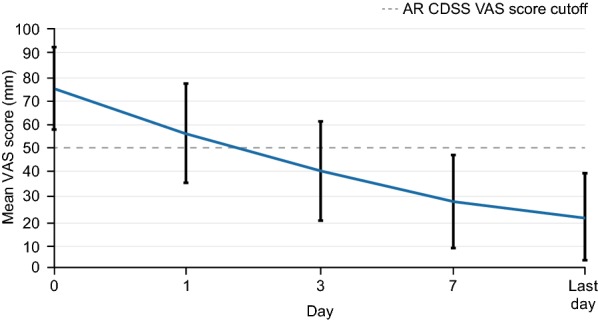
Effect of MP-AzeFlu on visual analogue scale (VAS) score over time. Data presented as mean and standard deviation. *AR* allergic rhinitis, *CDSS* clinical decision support system, *MP*-*AzeFlu*, a novel formulation of an intranasal antihistamine, azelastine hydrochloride, and an intranasal corticosteroid, fluticasone propionate, in a single spray (Reprinted with permission from Klimek et al. [126])

The efficacy of MP-AzeFlu in real life has been assessed in a pan-European noninterventional study conducted in Germany, Scandinavia (Sweden, Norway, and Denmark), the United Kingdom, and Romania [123, 124, 126, 127], using a VAS in line with EU and MACVIA-ARIA initiatives. In these studies, the VAS question was clearly stated: “please reflect on how bothersome your symptoms were within the previous 24 h”, and text anchors provided from 0 (not at all bothersome) to 100 (extremely bothersome). Data collected during the German arm of the study highlighted the suboptimal symptom relief provided by frequently prescribed AR medications, including antihistamines, INSs, and multiple therapies [126]. Patients had an average baseline VAS score of 75.4 mm, despite the fact that almost all of them were receiving AR treatment, and almost two-thirds of that group were receiving multiple-therapy regimens [126]. Uncontrolled disease was also apparent from high physician consultation rates, with patients averaging 2.6 AR visits in the current calendar year. MP-AzeFlu reduced VAS scores from 75.4 mm at baseline to 21.3 mm by the last visit, a clinically relevant shift of 54.1 mm (Fig. 5) [126]. Patients treated with MP-AzeFlu achieved the AR CDSS control VAS score cutoff of 50 mm within 3 days. One of every two patients felt his or her symptoms were well controlled at day 3. Effectiveness was confirmed irrespective of phenotype (SAR, PAR, or both), age (12–17 years, 18–65 years, and > 65 years), and symptom severity at baseline [126].

The efficacy results from Germany were later confirmed in patients from other countries in this pan-European study. Treatment with MP-AzeFlu significantly reduced the mean VAS score from 73.7 mm at baseline to 23.4 mm by the last visit and the results were consistent across countries, age, phenotype, and degrees of disease severity [145].

MP-AzeFlu was well tolerated in real-life studies, with dysgeusia as the most frequently reported adverse drug reaction [145]. Nausea, sneezing, nasal discomfort rhinorrhea, application site pain, and epistaxis were also reported by some patients; none of the adverse events or adverse drug reactions were considered serious [145].

#### Immunotherapy for AR uncontrolled by pharmacologic agents

For patients with AR whose symptoms remain uncontrolled by pharmacotherapies, use of allergen immunotherapy may be an important treatment option [26]. Allergen-specific immunotherapies involve administering allergen extract, either subcutaneously or sublingually, to desensitize patients against specific allergens and reduce symptoms and medication requirements [26]. Both subcutaneous immunotherapy (SCIT) and sublingual immunotherapy (SLIT) have been shown to be effective in patients with SAR and perennial AR, and are well tolerated with localised side effects, such as pain, itching, and swelling [146]. Because subcutaneous administration of allergens can occasionally cause severe allergic reactions requiring access to adrenaline and other resuscitative measures, SCIT should be administered in a specialist clinic, whereas SLIT can be self-administered [146, 147]. Indirect comparisons indicate that both SLIT and SCIT may be as potent as pharmacotherapy in controlling symptoms of AR [148, 149].

## Conclusions

MP-AzeFlu is currently the only combination therapy available for the treatment of AR. MP-AzeFlu blocks two important pathophysiological pathways involved in the early- and late-phase reactions in the disease, providing rapid relief from all symptoms associated with AR. However, all patients are not the same, and the inflammatory pattern may vary considerably depending on what drives the disease in each individual. The new AR CDSS algorithm and use of the VAS can support management of AR in both primary and secondary care. In addition, the effectiveness of combination treatment compared with corticosteroids alone should be evaluated in broader, real-life populations, including patients with both AR and NAR, as well as those with concomitant rhinosinusitis. Further mechanism of action studies will also pinpoint more exactly the pathophysiological gaps that are plugged with MP-AzeFlu therapy.

Another question for future research is whether it might be beneficial to block additional pathophysiological pathways that are not covered by antihistamine or corticosteroid treatment. CysLTs such as LTC_4_ and LTD_4_ are proinflammatory mediators and potent bronchoconstrictors that induce nasal congestion. Combining an anti-leukotriene with an antihistamine has proven to be effective in some patients with AR [150, 151], especially those with concomitant asthma and rhinitis. To understand the best principles of disease control, it is necessary to explore important biomarkers and evaluation parameters that relate to optimal disease control in both the short and long term. This calls for better clinical evaluation instruments that take into account the complexity of a disease with many different faces.

## Abbreviations

AR: allergic rhinitis
ARIA: allergic rhinitis and its impact on asthma
AZE: azelastine hydrochloride
CDSS: clinical decision support system
CysLT: cysteinyl leukotriene
EU: European Union
FP: fluticasone propionate
GM-CSF: granulocyte macrophage colony-stimulating factor
ICAM-1: intercellular adhesion molecule-1
IgE: immunoglobulin E
IL: interleukin
INAH: intranasal antihistamine
INS: intranasal corticosteroid
ITT: intent to treat
LS: least squares
LT: leukotriene
LTRA: leukotriene receptor antagonist
MACVIA: Contre les MAladies Chroniques pour un VIeillissement Actif
NAR: non-allergic rhinitis
NIS: noninterventional study
OAH: oral antihistamine
PAR: perennial allergic rhinitis
PP: per protocol
QD: once daily
QoL: quality of life
RQLQ: Rhinitis Quality of Life Questionnaire
rT7SS: reflective total of seven symptom scores
rTNSS: reflective total nasal symptom score
rTOSS: reflective total ocular symptom score
SAR: seasonal allergic rhinitis
TNF: tumour necrosis factor
VAS: visual analogue scale

## References

[1] Eriksson J, Ekerljung L, Ronmark E, Dahlen B, Ahlstedt S, Dahlen SE (2012). Update of prevalence of self-reported allergic rhinitis and chronic nasal symptoms among adults in Sweden. Clin Respir J.

[2] Mullol J (2009). A survey of the burden of allergic rhinitis in Spain. J Investig Allergol Clin Immunol.

[3] Canonica GW, Mullol J, Pradalier A, Didier A (2008). Patient perceptions of allergic rhinitis and quality of life: findings from a survey conducted in Europe and the United States. World Allergy Organ J.

[4] Brozek JL, Bousquet J, Agache I, Agarwal A, Bachert C, Bosnic-Anticevich S (2017). Allergic rhinitis and its impact on asthma (ARIA) guidelines—2016 revision. J Allergy Clin Immunol.

[5] Brozek JL, Bousquet J, Baena-Cagnani CE, Bonini S, Canonica GW, Casale TB (2010). Allergic rhinitis and its impact on asthma (ARIA) guidelines: 2010 revision. J Allergy Clin Immunol.

[6] Canonica GW, Bousquet J, Mullol J, Scadding GK, Virchow JC (2007). A survey of the burden of allergic rhinitis in Europe. Allergy.

[7] Nathan RA (2007). The burden of allergic rhinitis. Allergy Asthma Proc.

[8] Valovirta E, Myrseth SE, Palkonen S (2008). The voice of the patients: allergic rhinitis is not a trivial disease. Curr Opin Allergy Clin Immunol.

[9] Walker S, Khan-Wasti S, Fletcher M, Cullinan P, Harris J, Sheikh A (2007). Seasonal allergic rhinitis is associated with a detrimental effect on examination performance in United Kingdom teenagers: case–control study. J Allergy Clin Immunol.

[10] Benninger MS, Benninger RM (2009). The impact of allergic rhinitis on sexual activity, sleep, and fatigue. Allergy Asthma Proc.

[11] Braido F, Baiardini I, Scichilone N, Musarra A, Menoni S, Ridolo E (2014). Illness perception, mood and coping strategies in allergic rhinitis: are there differences among ARIA classes of severity?. Rhinology.

[12] Vuurman EF, Vuurman LL, Lutgens I, Kremer B (2014). Allergic rhinitis is a risk factor for traffic safety. Allergy.

[13] Cardell LO, Olsson P, Andersson M, Welin KO, Svensson J, Tennvall GR (2016). TOTALL: high cost of allergic rhinitis—a national Swedish population-based questionnaire study. NPJ Prim Care Respir Med.

[14] Lamb CE, Ratner PH, Johnson CE, Ambegaonkar AJ, Joshi AV, Day D (2006). Economic impact of workplace productivity losses due to allergic rhinitis compared with select medical conditions in the United States from an employer perspective. Curr Med Res Opin.

[15] Bousquet J, Malva J, Nogues M, Manas LR, Vellas B, Farrell J (2015). Operational definition of active and healthy aging (AHA): the European innovation partnership (EIP) on AHA reference site questionnaire: Montpellier October 20–21, 2014, Lisbon July 2, 2015. J Am Med Dir Assoc.

[16] Demoly P, Bousquet PJ, Mesbah K, Bousquet J, Devillier P (2013). Visual analogue scale in patients treated for allergic rhinitis: an observational prospective study in primary care: asthma and rhinitis. Clin Exp Allergy.

[17] Bousquet PJ, Demoly P, Devillier P, Mesbah K, Bousquet J (2013). Impact of allergic rhinitis symptoms on quality of life in primary care. Int Arch Allergy Immunol.

[18] Bousquet J, Annesi-Maesano I, Carat F, Leger D, Rugina M, Pribil C (2005). Characteristics of intermittent and persistent allergic rhinitis: DREAMS study group. Clin Exp Allergy.

[19] Maurer M, Zuberbier T (2007). Undertreatment of rhinitis symptoms in Europe: findings from a cross-sectional questionnaire survey. Allergy.

[20] Bernstein JA (2010). Allergic and mixed rhinitis: epidemiology and natural history. Allergy Asthma Proc.

[21] Bousquet J, Bachert C, Canonica GW, Casale TB, Cruz AA, Lockey RJ (2009). Unmet needs in severe chronic upper airway disease (SCUAD). J Allergy Clin Immunol.

[22] Bousquet PJ, Bachert C, Canonica GW, Casale TB, Mullol J, Klossek JM (2010). Uncontrolled allergic rhinitis during treatment and its impact on quality of life: a cluster randomized trial. J Allergy Clin Immunol.

[23] Rondon C, Campo P, Togias A, Fokkens WJ, Durham SR, Powe DG (2012). Local allergic rhinitis: concept, pathophysiology, and management. J Allergy Clin Immunol.

[24] Rondon C, Campo P, Zambonino MA, Blanca-Lopez N, Torres MJ, Melendez L (2014). Follow-up study in local allergic rhinitis shows a consistent entity not evolving to systemic allergic rhinitis. J Allergy Clin Immunol.

[25] Rondon C, Campo P, Eguiluz-Gracia I, Plaza C, Bogas G, Galindo P (2018). Local allergic rhinitis is an independent rhinitis phenotype: the results of a 10-year follow-up study. Allergy.

[26] Bousquet J, Khaltaev N, Cruz AA, Denburg J, Fokkens WJ, Togias A (2008). Allergic rhinitis and its impact on asthma (ARIA) 2008 update (in collaboration with the World Health Organization, GA(2)LEN and AllerGen). Allergy.

[27] Okubo K, Nakashima M, Miyake N, Komatsubara M, Okuda M (2009). Comparison of fluticasone furoate and fluticasone propionate for the treatment of Japanese cedar pollinosis. Allergy Asthma Proc.

[28] Mandl M, Nolop K, Lutsky BN (1997). Comparison of once daily mometasone furoate (Nasonex) and fluticasone propionate aqueous nasal sprays for the treatment of perennial rhinitis. 194-079 Study Group. Ann Allergy Asthma Immunol.

[29] Meltzer EO, Blaiss MS, Naclerio RM, Stoloff SW, Derebery MJ, Nelson HS (2012). Burden of allergic rhinitis: allergies in America, Latin America, and Asia-Pacific adult surveys. Allergy Asthma Proc.

[30] Nathan RA, Meltzer EO, Derebery J, Campbell UB, Stang PE, Corrao MA (2008). The prevalence of nasal symptoms attributed to allergies in the United States: findings from the burden of rhinitis in an America survey. Allergy Asthma Proc.

[31] Dymista summary of product characteristics for Norway. https://www.medicines.org.uk/emc/medicine/27579. 2014. Accessed 12 Oct 2018.

[32] Bousquet J, Schunemann H, Arnavielhe S, Bachert C, Bedbrook A, Bergmann KC (2016). MACVIA clinical decision algorithm in allergic rhinitis in adolescents and adults. J Allergy Clin Immunol.

[33] Plaza MV (2015). GEMA(4.0). Guidelines for asthma management. Arch Bronconeumol.

[34] Price D, Scadding G, Ryan D, Bachert C, Canonica GW, Mullol J (2015). The hidden burden of adult allergic rhinitis: UK healthcare resource utilisation survey. Clin Transl Allergy.

[35] Sin B, Togias A (2011). Pathophysiology of allergic and nonallergic rhinitis. Proc Am Thorac Soc.

[36] Min YG (2010). The pathophysiology, diagnosis and treatment of allergic rhinitis. Allergy Asthma Immunol Res.

[37] Thompson A, Sardana N, Craig TJ (2013). Sleep impairment and daytime sleepiness in patients with allergic rhinitis: the role of congestion and inflammation. Ann Allergy Asthma Immunol.

[38] Stull DE, Roberts L, Frank L, Heithoff K (2007). Relationship of nasal congestion with sleep, mood, and productivity. Curr Med Res Opin.

[39] Valero A, Munoz-Cano R, Sastre J, Navarro AM, Marti-Guadano E, Davila I (2012). The impact of allergic rhinitis on symptoms, and quality of life using the new criterion of ARIA severity classification. Rhinology.

[40] Bellanti JA, Settipane RA (2012). The burden of allergic rhinitis on patients’ quality of life. Allergy Asthma Proc.

[41] Small M, Piercy J, Demoly P, Marsden H (2013). Burden of illness and quality of life in patients being treated for seasonal allergic rhinitis: a cohort survey. Clin Transl Allergy.

[42] Meltzer EO, Blaiss MS, Derebery MJ, Mahr TA, Gordon BR, Sheth KK (2009). Burden of allergic rhinitis: results from the pediatric allergies in America survey. J Allergy Clin Immunol.

[43] Meltzer EO, Gross GN, Katial R, Storms WW (2012). Allergic rhinitis substantially impacts patient quality of life: findings from the Nasal Allergy Survey Assessing Limitations. J Fam Pract.

[44] de la Hoz Caballer B, Rodriguez M, Fraj J, Cerecedo I, Antolin-Amerigo D, Colas C (2012). Allergic rhinitis and its impact on work productivity in primary care practice and a comparison with other common diseases: the cross-sectional study to evaluate work productivity in allergic rhinitis compared with other common diseases (CAPRI) study. Am J Rhinol Allergy.

[45] Craig TJ, Sherkat A, Safaee S (2010). Congestion and sleep impairment in allergic rhinitis. Curr Allergy Asthma Rep.

[46] Michels Dde S, Rodrigues Ada M, Nakanishi M, Sampaio AL, Venosa AR (2014). Nasal involvement in obstructive sleep apnea syndrome. Int J Otolaryngol.

[47] Ferguson BJ (2004). Influences of allergic rhinitis on sleep. Otolaryngol Head Neck Surg.

[48] Peters-Golden M, Gleason MM, Togias A (2006). Cysteinyl leukotrienes: multi-functional mediators in allergic rhinitis. Clin Exp Allergy.

[49] Ramires R, Caiaffa MF, Tursi A, Haeggstrom JZ, Macchia L (2004). Novel inhibitory effect on 5-lipoxygenase activity by the anti-asthma drug montelukast. Biochem Biophys Res Commun.

[50] Anderson R, Theron AJ, Gravett CM, Steel HC, Tintinger GR, Feldman C (2009). Montelukast inhibits neutrophil pro-inflammatory activity by a cyclic AMP-dependent mechanism. Br J Pharmacol.

[51] Woszczek G, Chen LY, Alsaaty S, Nagineni S, Shelhamer JH (2010). Concentration-dependent noncysteinyl leukotriene type 1 receptor-mediated inhibitory activity of leukotriene receptor antagonists. J Immunol.

[52] Tahan F, Jazrawi E, Moodley T, Rovati GE, Adcock IM (2008). Montelukast inhibits tumour necrosis factor-alpha-mediated interleukin-8 expression through inhibition of nuclear factor-kappaB p65-associated histone acetyltransferase activity. Clin Exp Allergy.

[53] Robinson AJ, Kashanin D, O’Dowd F, Williams V, Walsh GM (2008). Montelukast inhibition of resting and GM-CSF-stimulated eosinophil adhesion to VCAM-1 under flow conditions appears independent of cysLT(1)R antagonism. J Leukoc Biol.

[54] Ichiyama T, Hasegawa S, Umeda M, Terai K, Matsubara T, Furukawa S (2003). Pranlukast inhibits NF-kappa B activation in human monocytes/macrophages and T cells. Clin Exp Allergy.

[55] Fang SH, Yuan YM, Peng F, Li CT, Zhang LH, Lu YB (2009). Pranlukast attenuates ischemia-like injury in endothelial cells via inhibiting reactive oxygen species production and nuclear factor-kappaB activation. J Cardiovasc Pharmacol.

[56] Meliton AY, Munoz NM, Leff AR (2007). Blockade of avidity and focal clustering of beta 2-integrin by cysteinyl leukotriene antagonism attenuates eosinophil adhesion. J Allergy Clin Immunol.

[57] Weimer LK, Gamache DA, Yanni JM (1998). Histamine-stimulated cytokine secretion from human conjunctival epithelial cells: inhibition by the histamine H1 antagonist emedastine. Int Arch Allergy Immunol.

[58] Dobashi K, Iizuka K, Houjou S, Sakai H, Watanabe K, Mori M (1996). Effect of cetirizine on antigen-induced tracheal contraction of passively sensitized guinea pigs. Ann Allergy Asthma Immunol.

[59] Anthes JC, Gilchrest H, Richard C, Eckel S, Hesk D, West RE (2002). Biochemical characterization of desloratadine, a potent antagonist of the human histamine H(1) receptor. Eur J Pharmacol.

[60] Kanei A, Asano K, Kanai K, Furuta A, Sasaki K, Suzaki H (2014). Inhibitory action of levocetirizine on the production of eosinophil chemoattractants RANTES and eotaxin in vitro and in vivo. In Vivo.

[61] Kusters S, Schuligoi R, Huttenbrink KB, Rudert J, Wachs A, Szelenyi I (2002). Effects of antihistamines on leukotriene and cytokine release from dispersed nasal polyp cells. Arzneimittelforschung.

[62] Canonica GW, Blaiss M (2011). Antihistaminic, anti-inflammatory, and antiallergic properties of the nonsedating second-generation antihistamine desloratadine: a review of the evidence. World Allergy Organ J.

[63] Molimard M, Diquet B, Benedetti MS (2004). Comparison of pharmacokinetics and metabolism of desloratadine, fexofenadine, levocetirizine and mizolastine in humans. Fundam Clin Pharmacol.

[64] Urien S, Tillement JP, Ganem B, Kuch MD (1999). A pharmacokinetic-pharmacodynamic modelling of the antihistaminic (H1) effects of cetirizine. Int J Clin Pharmacol Ther.

[65] Horak F, Zieglmayer UP (2009). Azelastine nasal spray for the treatment of allergic and nonallergic rhinitis. Expert Rev Clin Immunol.

[66] Lytinas M, Kempuraj D, Huang M, Kandere K, Boucher W, Letourneau R (2002). Azelastine’s inhibition of histamine and tryptase release from human umbilical cord blood-derived cultured mast cells as well as rat skin mast cell-induced vascular permeability: comparison with olopatadine. Allergy Asthma Proc.

[67] Tamaoki J, Yamawaki I, Tagaya E, Kondo M, Aoshiba K, Nakata J (1999). Effect of azelastine on platelet-activating factor-induced microvascular leakage in rat airways. Am J Physiol.

[68] Van Hoecke H, Vandenbulcke L, Van Cauwenberge P (2007). Histamine and leukotriene receptor antagonism in the treatment of allergic rhinitis: an update. Drugs..

[69] Kempuraj D, Huang M, Kandere-Grzybowska K, Basu S, Boucher W, Letourneau R (2003). Azelastine inhibits secretion of IL-6, TNF-alpha and IL-8 as well as NF-kappaB activation and intracellular calcium ion levels in normal human mast cells. Int Arch Allergy Immunol.

[70] Hamasaki Y, Shafigeh M, Yamamoto S, Sato R, Zaitu M, Muro E (1996). Inhibition of leukotriene synthesis by azelastine. Ann Allergy Asthma Immunol.

[71] Matsuo S, Takayama S (1998). Influence of the anti-allergic agent, azelastine, on tumor necrosis factor-alpha (TNF-alpha) secretion from cultured mouse mast cells. In Vivo.

[72] Yoneda K, Yamamoto T, Ueta E, Osaki T (1997). Suppression by azelastine hydrochloride of NF-kappa B activation involved in generation of cytokines and nitric oxide. Jpn J Pharmacol.

[73] Ito H, Nakamura Y, Takagi S, Sakai K (1998). Effects of azelastine on the level of serum interleukin-4 and soluble CD23 antigen in the treatment of nasal allergy. Arzneimittelforschung.

[74] Ciprandi G, Pronzato C, Passalacqua G, Ricca V, Grogen J, Mela GS (1996). Topical azelastine reduces eosinophil activation and intercellular adhesion molecule-1 expression on nasal epithelial cells: an antiallergic activity. J Allergy Clin Immunol.

[75] Schumacher S, Kietzmann M, Stark H, Baumer W (2014). Unique immunomodulatory effects of azelastine on dendritic cells in vitro. Naunyn Schmiedebergs Arch Pharmacol.

[76] Holm A, Dijkstra M, Kleinjan A, Severijnen LA, Boks S, Mulder P (2001). Fluticasone propionate aqueous nasal spray reduces inflammatory cells in unchallenged allergic nasal mucosa: effects of single allergen challenge. J Allergy Clin Immunol.

[77] Alvarado-Valdes CA, Blomgren J, Weiler D, Gleich GJ, Reed CE, Field EA (1997). The effect of fluticasone propionate aqueous nasal spray on eosinophils and cytokines in nasal secretions of patients with ragweed allergic rhinitis. Clin Ther.

[78] Weido AJ, Reece LM, Alam R, Cook CK, Sim TC (1996). Intranasal fluticasone propionate inhibits recovery of chemokines and other cytokines in nasal secretions in allergen-induced rhinitis. Ann Allergy Asthma Immunol.

[79] Rak S, Jacobson MR, Sudderick RM, Masuyama K, Juliusson S, Kay AB (1994). Influence of prolonged treatment with topical corticosteroid (fluticasone propionate) on early and late phase nasal responses and cellular infiltration in the nasal mucosa after allergen challenge. Clin Exp Allergy.

[80] Ciprandi G, Ricca V, Passalacqua G, Fasolo A, Canonica GW (1998). Intranasal fluticasone propionate reduces ICAM-1 on nasal epithelial cells both during early and late phase after allergen challenge. Clin Exp Allergy.

[81] Silvestri M, Sabatini F, Scarso L, Cordone A, Dasic G, Rossi GA (2002). Fluticasone propionate downregulates nasal fibroblast functions involved in airway inflammation and remodeling. Int Arch Allergy Immunol.

[82] Erin EM, Zacharasiewicz AS, Nicholson GC, Tan AJ, Higgins LA, Williams TJ (2005). Topical corticosteroid inhibits interleukin-4, -5 and -13 in nasal secretions following allergen challenge. Clin Exp Allergy.

[83] Zhang N, Van Crombruggen K, Holtappels G, Lan F, Katotomichelakis M, Zhang L (2014). Suppression of cytokine release by fluticasone furoate vs. mometasone furoate in human nasal tissue ex vivo. PLoS ONE.

[84] Di Lorenzo G, Pacor ML, Pellitteri ME, Gangemi S, Di Blasi P, Candore G (2002). In vitro effects of fluticasone propionate on IL-13 production by mitogen-stimulated lymphocytes. Mediators Inflamm.

[85] Ohnishi M, Takizawa R, Yokoshima K, Okubo K, Okuda M, Yagi T (1995). Generation of tumor necrosis factor alpha by human nasal epithelial cells and inhibition by fluticasone propionate. Arerugi.

[86] Ohnishi M, Takizawa R, Ohkubo K, Yokosima K, Okuda M, Yagi T (1994). Fluticasone propionate reduced the production of GM-CSF, IL-6 and IL-8 generated from cultured nasal epithelial cells. Arerugi.

[87] Wandalsen GF, Mendes AI, Sole D (2010). Objective improvement in nasal congestion and nasal hyperreactivity with use of nasal steroids in persistent allergic rhinitis. Am J Rhinol Allergy.

[88] Kanaoka Y, Boyce JA (2014). Cysteinyl leukotrienes and their receptors; emerging concepts. Allergy Asthma Immunol Res.

[89] Marshall GD (2000). Therapeutic options in allergic disease: antihistamines as systemic antiallergic agents. J Allergy Clin Immunol.

[90] Mygind N, Nielsen LP, Hoffmann HJ, Shukla A, Blumberga G, Dahl R (2001). Mode of action of intranasal corticosteroids. J Allergy Clin Immunol.

[91] Carr WW, Ratner P, Munzel U, Murray R, Price D, Canonica GW (2012). Comparison of intranasal azelastine to intranasal fluticasone propionate for symptom control in moderate-to-severe seasonal allergic rhinitis. Allergy Asthma Proc.

[92] Chen ST, Lu KH, Sun HL, Chang WT, Lue KH, Chou MC (2006). Randomized placebo-controlled trial comparing montelukast and cetirizine for treating perennial allergic rhinitis in children aged 2–6 yr. Pediatr Allergy Immunol.

[93] Alevizos M, Karagkouni A, Vasiadi M, Sismanopoulos N, Makris M, Kalogeromitros D (2013). Rupatadine inhibits inflammatory mediator release from human laboratory of allergic diseases 2 cultured mast cells stimulated by platelet-activating factor. Ann Allergy Asthma Immunol.

[94] Maiti R, Rahman J, Jaida J, Allala U, Palani A (2010). Rupatadine and levocetirizine for seasonal allergic rhinitis: a comparative study of efficacy and safety. Arch Otolaryngol Head Neck Surg.

[95] Marmouz F, Giralt J, Izquierdo I (2011). Morning and evening efficacy evaluation of rupatadine (10 and 20 mg), compared with cetirizine 10 mg in perennial allergic rhinitis: a randomized, double-blind, placebo-controlled trial. J Asthma Allergy.

[96] Maiti R, Jaida J, Rahman J, Gaddam R, Palani A (2011). Olopatadine hydrochloride and rupatadine fumarate in seasonal allergic rhinitis: a comparative study of efficacy and safety. J Pharmacol Pharmacother.

[97] Berger W, Hampel F, Bernstein J, Shah S, Sacks H, Meltzer EO (2006). Impact of azelastine nasal spray on symptoms and quality of life compared with cetirizine oral tablets in patients with seasonal allergic rhinitis. Ann Allergy Asthma Immunol.

[98] Keith PK, Scadding GK (2009). Are intranasal corticosteroids all equally consistent in managing ocular symptoms of seasonal allergic rhinitis?. Curr Med Res Opin.

[99] Bielory L, Katelaris CH, Lightman S, Naclerio RM (2007). Treating the ocular component of allergic rhinoconjunctivitis and related eye disorders. MedGenMed.

[100] Hong J, Bielory B, Rosenberg JL, Bielory L (2011). Efficacy of intranasal corticosteroids for the ocular symptoms of allergic rhinitis: a systematic review. Allergy Asthma Proc.

[101] Bielory L (2008). Intranasal corticosteroids and the eye: from negative ocular effects to clinical efficacy as a class effect. Ann Allergy Asthma Immunol.

[102] Origlieri C, Bielory L (2009). Intranasal corticosteroids: do they improve ocular allergy?. Curr Allergy Asthma Rep.

[103] Scadding GK, Keith PK (2008). Fluticasone furoate nasal spray consistently and significantly improves both the nasal and ocular symptoms of seasonal allergic rhinitis: a review of the clinical data. Expert Opin Pharmacother.

[104] Maspero JF, Walters RD, Wu W, Philpot EE, Naclerio RM, Fokkens WJ (2010). An integrated analysis of the efficacy of fluticasone furoate nasal spray on individual nasal and ocular symptoms of seasonal allergic rhinitis. Allergy Asthma Proc.

[105] Rodrigo GJ, Neffen H (2011). Efficacy of fluticasone furoate nasal spray vs. placebo for the treatment of ocular and nasal symptoms of allergic rhinitis: a systematic review. Clin Exp Allergy.

[106] Ratner P, Van Bavel J, Mohar D, Jacobs RL, Hampel F, Howland W (2015). Efficacy of daily intranasal fluticasone propionate on ocular symptoms associated with seasonal allergic rhinitis. Ann Allergy Asthma Immunol.

[107] Bielory L (2002). Role of antihistamines in ocular allergy. Am J Med.

[108] Hellings PW, Fokkens WJ, Akdis C, Bachert C, Cingi C, Dietz de Loos D (2013). Uncontrolled allergic rhinitis and chronic rhinosinusitis: where do we stand today?. Allergy.

[109] Esteban CA, Klein RB, Kopel SJ, McQuaid EL, Fritz GK, Seifer R (2014). Underdiagnosed and undertreated allergic rhinitis in urban school-aged children with asthma. Pediatr Allergy Immunol Pulmonol.

[110] Schatz M (2007). A survey of the burden of allergic rhinitis in the USA. Allergy.

[111] Anolik R, Mometasone Furoate Nasal Spray With Loratadine Study G (2008). Clinical benefits of combination treatment with mometasone furoate nasal spray and loratadine vs monotherapy with mometasone furoate in the treatment of seasonal allergic rhinitis. Ann Allergy Asthma Immunol.

[112] Ratner PH, van Bavel JH, Martin BG, Hampel FC, Howland WC, Rogenes PR (1998). A comparison of the efficacy of fluticasone propionate aqueous nasal spray and loratadine, alone and in combination, for the treatment of seasonal allergic rhinitis. J Fam Pract.

[113] Di Lorenzo G, Pacor ML, Pellitteri ME, Morici G, Di Gregoli A, Lo Bianco C (2004). Randomized placebo-controlled trial comparing fluticasone aqueous nasal spray in mono-therapy, fluticasone plus cetirizine, fluticasone plus montelukast and cetirizine plus montelukast for seasonal allergic rhinitis. Clin Exp Allergy.

[114] Ratner PH, Hampel F, Van Bavel J, Amar NJ, Daftary P, Wheeler W (2008). Combination therapy with azelastine hydrochloride nasal spray and fluticasone propionate nasal spray in the treatment of patients with seasonal allergic rhinitis. Ann Allergy Asthma Immunol.

[115] D’Addio A, Ruiz N, Mayer M, Murray R, Bachert C (2015). Deposition characteristics of a new allergic rhinitis nasal spray (MP29-02*) in an anatomical model of the human nasal cavity. Clin Transl Allergy..

[116] D'Addio AD, Ruiz NM, Mayer MJ, Berger W, Meltzer EO. Quantification of the distribution of MP29-02 (dymista-azelastine HCl/fluticasone propionate nasal spray) in an anatomical model of the human nasal cavity. In: 2015 AAAAI Annual Meeting 20–24 February 2015, Houston, TX.

[117] Bousquet J, Bachert C, Bernstein J, Canonica GW, Carr W, Dahl R (2015). Advances in pharmacotherapy for the treatment of allergic rhinitis; MP29-02 (a novel formulation of azelastine hydrochloride and fluticasone propionate in an advanced delivery system) fills the gaps. Expert Opin Pharmacother.

[118] Meltzer E, Ratner P, Bachert C, Carr W, Berger W, Canonica GW (2013). Clinically relevant effect of a new intranasal therapy (MP29-02) in allergic rhinitis assessed by responder analysis. Int Arch Allergy Immunol.

[119] Roca-Ferrer J, Pujols L, Perez-Gonzalez M, Alobid I, Valero A, Picado C (2015). MP29-02 reduces both eosinophil survival induced by epithelial cell secretions from nasal mucosa. Clin Transl Allergy.

[120] Berger W, Bousquet J, Fox AT, Just J, Muraro A, Nieto A (2016). MP-AzeFlu is more effective than fluticasone propionate for the treatment of allergic rhinitis in children. Allergy.

[121] Carr W, Bernstein J, Lieberman P, Meltzer E, Bachert C, Price D (2012). A novel intranasal therapy of azelastine with fluticasone for the treatment of allergic rhinitis. J Allergy Clin Immunol.

[122] Derendorf H, Munzel U, Petzold U, Maus J, Mascher H, Hermann R (2012). Bioavailability and disposition of azelastine and fluticasone propionate when delivered by MP29-02, a novel aqueous nasal spray. Br J Clin Pharmacol.

[123] Dollner R, Lorentz LP, Sheyauldeen S, Kuhl H, Steinsvag S. Real-life effectiveness of a new allergic rhinitis therapy (MP29-02) in Norway. Allergy. 2015;70(502).

[124] Haahr P, Jacobsen C, Blegvad S, Christensen M, Kuhl H, Nielsen K. Real life effectiveness of a new allergic rhinitis therapy (MP29-02) in Denmark. Allergy. 2015;70(500).

[125] Hampel FC, Ratner PH, Van Bavel J, Amar NJ, Daftary P, Wheeler W (2010). Double-blind, placebo-controlled study of azelastine and fluticasone in a single nasal spray delivery device. Ann Allergy Asthma Immunol.

[126] Klimek L, Bachert C, Mosges R, Munzel U, Price D, Virchow JC (2015). Effectiveness of MP29-02 for the treatment of allergic rhinitis in real-life: results from a noninterventional study. Allergy Asthma Proc.

[127] Stjarne P, Strand V, Theman K, Kuhl H, Ehngage A (2015). Real-life effectiveness of a new allergic rhinitis therapy (MP29-02) in Sweden. Clin Transl Allergy..

[128] Berger WE, Shah S, Lieberman P, Hadley J, Price D, Munzel U (2014). Long-term, randomized safety study of MP29-02 (a novel intranasal formulation of azelastine hydrochloride and fluticasone propionate in an advanced delivery system) in subjects with chronic rhinitis. J Allergy Clin Immunol Pract.

[129] Price D, Shah S, Bhatia S, Bachert C, Berger W, Bousquet J (2013). A new therapy (MP29-02) is effective for the long-term treatment of chronic rhinitis. J Investig Allergol Clin Immunol.

[130] Berger W, Meltzer EO, Amar N, Fox AT, Just J, Muraro A (2016). Efficacy of MP-AzeFlu in children with seasonal allergic rhinitis: importance of paediatric symptom assessment. Pediatr Allergy Immunol.

[131] van As A, Bronsky E, Grossman J, Meltzer E, Ratner P, Reed C (1991). Dose tolerance study of fluticasone propionate aqueous nasal spray in patients with seasonal allergic rhinitis. Ann Allergy.

[132] Salapatek AM, Lee J, Patel D, D’Angelo P, Liu J, Zimmerer RO (2011). Solubilized nasal steroid (CDX-947) when combined in the same solution nasal spray with an antihistamine (CDX-313) provides improved, fast-acting symptom relief in patients with allergic rhinitis. Allergy Asthma Proc.

[133] Berger WE, Meltzer EO (2015). Intranasal spray medications for maintenance therapy of allergic rhinitis. Am J Rhinol Allergy.

[134] Meltzer EO, LaForce C, Ratner P, Price D, Ginsberg D, Carr W (2012). MP29-02 (a novel intranasal formulation of azelastine hydrochloride and fluticasone propionate) in the treatment of seasonal allergic rhinitis: a randomized, double-blind, placebo-controlled trial of efficacy and safety. Allergy Asthma Proc.

[135] Dymista approved in children 6 to 11 years of age with seasonal allergic rhinitis. http://cisn.co/2AD11JK. Accessed 12 Oct 2018.

[136] Bousquet J, Clark TJ, Hurd S, Khaltaev N, Lenfant C, O’Byrne P (2007). GINA guidelines on asthma and beyond. Allergy.

[137] Bousquet J, Schunemann HJ, Fonseca J, Samolinski B, Bachert C, Canonica GW (2015). MACVIA-ARIA Sentinel NetworK for allergic rhinitis (MASK-rhinitis): the new generation guideline implementation. Allergy.

[138] Bousquet PJ, Combescure C, Klossek JM, Daures JP, Bousquet J (2009). Change in visual analog scale score in a pragmatic randomized cluster trial of allergic rhinitis. J Allergy Clin Immunol.

[139] Bousquet PJ, Combescure C, Neukirch F, Klossek JM, Mechin H, Daures JP (2007). Visual analog scales can assess the severity of rhinitis graded according to ARIA guidelines. Allergy.

[140] Bousquet J, Bachert C, Canonica GW, Mullol J, Van Cauwenberge P, Jensen CB (2010). Efficacy of desloratadine in persistent allergic rhinitis - a GA(2)LEN study. Int Arch Allergy Immunol.

[141] Bousquet J, Bachert C, Canonica GW, Mullol J, Van Cauwenberge P, Bindslev Jensen C (2009). Efficacy of desloratadine in intermittent allergic rhinitis: a GA(2)LEN study. Allergy.

[142] Ratner PH, Howland WC, Arastu R, Philpot EE, Klein KC, Baidoo CA (2003). Fluticasone propionate aqueous nasal spray provided significantly greater improvement in daytime and nighttime nasal symptoms of seasonal allergic rhinitis compared with montelukast. Ann Allergy Asthma Immunol.

[143] Catana IV, Chirila M, Negoias S, Bologa R, Cosgarea M (2013). Effects of corticosteroids on hyposmia in persistent allergic rhinitis. Clujul Med.

[144] Yamamoto H, Yamada T, Sakashita M, Kubo S, Susuki D, Tokunaga T (2012). Efficacy of prophylactic treatment with montelukast and montelukast plus add-on loratadine for seasonal allergic rhinitis. Allergy Asthma Proc.

[145] Klimek L, Bachert C, Stjarne P, Dollner R, Larsen P, Haahr P (2016). MP-AzeFlu provides rapid and effective allergic rhinitis control in real life: a pan-European study. Allergy Asthma Proc.

[146] Durham SR, Penagos M (2016). Sublingual or subcutaneous immunotherapy for allergic rhinitis?. J Allergy Clin Immunol.

[147] Quirt J, Gagnon R, Ellis AK, Kim HL (2018). CSACI position statement: prescribing sublingual immunotherapy tablets for aeroallergens. Allergy Asthma Clin Immunol.

[148] Matricardi PM, Kuna P, Wahn U, Narkus A (2011). Subcutaneous immunotherapy and pharmacotherapy in seasonal allergic rhinitis: a comparison based on meta-analyses. J Allergy Clin Immunol.

[149] Durham SR, Creticos PS, Nelson HS, Li Z, Kaur A, Meltzer EO (2016). Treatment effect of sublingual immunotherapy tablets and pharmacotherapies for seasonal and perennial allergic rhinitis: pooled analyses. J Allergy Clin Immunol.

[150] Ciebiada M, Gorska-Ciebiada M, Barylski M, Kmiecik T, Gorski P (2011). Use of montelukast alone or in combination with desloratadine or levocetirizine in patients with persistent allergic rhinitis. Am J Rhinol Allergy.

[151] Adsule SM, Misra D (2010). Long term treatment with montelukast and levocetirizine combination in persistent allergic rhinitis: review of recent evidence. J Indian Med Assoc.

